# Supplementary material for: Surface Microbiome Profiling of Dental Elevators Using Third‐Generation Sequencing: Implications for Infection Control in Dental Practice
Source: Microbiologyopen. 2025 Dec 8;14(6):e70178. doi: 10.1002/mbo3.70178 (PMC12685768; doi:10.1002/mbo3.70178)
Supplement: Supplementary file 1 — customer_backup. [file MBO3-14-e70178-s001.zip › customer_backup/customer_backup/data_assement/M06_reads_length.pdf]

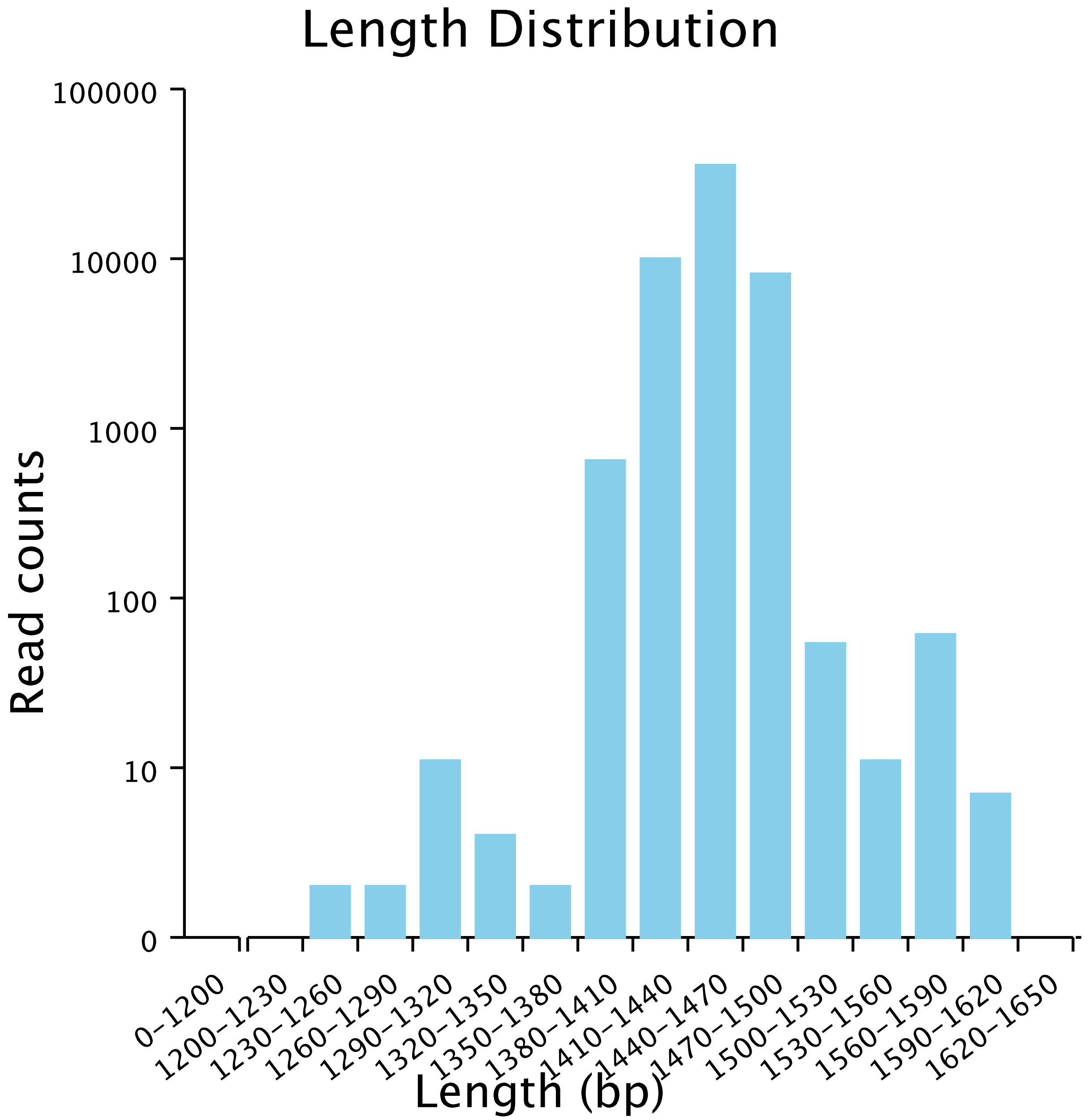
Length Distribution
Read counts
100000
10000
1000
100
10
0
0–1200
1200–1230
1230–1260
1260–1290
1290–1320
1320–1350
1350–1380
1380–1410
1410–1440
1440–1470
1470–1500
1500–1530
1530–1560
1560–1590
1590–1620
1620–1650
Length (bp)
